# Current practices in hemodynamic monitoring and management during non-cardiac surgery in Austria

**DOI:** 10.1186/s12871-025-03374-7

**Published:** 2025-09-23

**Authors:** C. Gaik, P. Paal, DA Reuter, H. Wulf, Benjamin Vojnar

**Affiliations:** 1 Marburg University, Marburg, Germany; 2 Department of Anesthesiology and Intensive Care Medicine, University Hospital Giessen and Marburg, Campus Marburg,, Marburg, Germany; 3 St. John of God Hospital, Paracelsus Medical University, Salzburg, Austria; 4 Department of Anesthesia and Intensive Care, University Hospital Rostock, Rostock, Germany

**Keywords:** Advanced hemodynamic monitoring, Hypotension, Perioperative medicine, Blood pressure management, Cardiac output

## Abstract

**Background:**

Intraoperative hemodynamic monitoring has advanced significantly over the past few decades, enhancing patient safety and improving perioperative outcomes. This survey aimed to examine current practices in intraoperative hemodynamic management in Austria.

**Method:**

Between January 2024 and February 2024, members of the Austrian Society of Anesthesiology, Resuscitation, and Intensive Care Medicine (ÖGARI) with a registered email address (*n* = 1,839) were invited to participate in an anonymous web-based survey.

**Results:**

A total of 201 questionnaires were received, of which 177 were fully completed. When using intermittent oscillometry, 40% (71/177) of respondents measure blood pressure every three minutes during anesthesia induction. Nearly 45% (80/177) routinely insert an arterial catheter before anesthesia induction, using mean arterial pressure (MAP) to Guide blood pressure management. While 36% (61/168) consider a MAP of 60 mmHg critically low, 48% (80/168) set the threshold at 65 mmHg. Intraoperative hypotension is predominantly managed at individual discretion by 79% (140/177), while 12% (21/177) follow institutional standardized protocols. A pulse contour analysis monitor is available in 94% (166/177) of respondents, with 49% (87/177) reporting frequent use. Regarding the limited use of advanced hemodynamic monitoring in high-risk non-cardiac surgery patients, 64% (113/177) perceived its added value as too low, while 57% (100/177) cite a lack of experience in interpreting the parameters as a barrier to implementation.

**Discussion:**

This survey among ÖGARI members provides key insights into intraoperative hemodynamic monitoring in Austrian hospitals. The findings suggest that respondents largely follow international recommendations, particularly concerning general blood pressure thresholds, measurement intervals, and indications for advanced hemodynamic monitoring. However, hemodynamic management appear to be only partially standardized, with decisions primarily left to the discretion of the anesthetist.

**Trial registration:**

The study was prospectively registered in the German Clinical Trials Register (DRKS; registration number DRKS00033181 on December 6, 2023).

**Supplementary Information:**

The online version contains supplementary material available at 10.1186/s12871-025-03374-7.

## Background

Basic hemodynamic monitoring is a key component of perioperative care and ensures patient safety. Advanced hemodynamic monitoring, including measuring cardiac output (CO) – the primary hemodynamic determinant of oxygen delivery – may provide critical insights into the underlying causes of hemodynamic instability and guide therapeutic interventions with fluids, vasopressors, and inotropes.

Over the past decades, numerous minimally invasive and non-invasive methods for advanced hemodynamic monitoring have been developed [1]. Consequently, highly invasive techniques, such as pulmonary artery catheterization or transpulmonary thermodilution, are nowadays rarely used in non-cardiac surgery patients. However, despite these advancements, intraoperative hemodynamic monitoring and management practices remain largely unclear. In addition, practical recommendations for intraoperative advanced hemodynamic monitoring were unavailable for a long time. This changed with the publication of the first German Guideline for intraoperative hemodynamic monitoring and management in adults undergoing non-cardiac surgery in late 2023 [2]. The publication of this guideline was accompanied by a survey exploring how anesthesiologists in Germany conducted intraoperative hemodynamic monitoring and management prior to its release [3]. With this survey, we aimed to investigate current practices in Austria to obtain a more comprehensive understanding of the German-speaking region.

## Materials and methods

### Ethics approval and setting

The study was prospectively registered in the German Clinical Trials Register (DRKS; registration number DRKS00033181). Registration was completed on December 6, 2023, and the first response was received on January 10, 2024. It was conducted in accordance with the principles of the Declaration of Helsinki, and ethical approval was obtained in advance from the Ethics Committee of the Medical Faculty at Philipps University Marburg (Reference: 23–275 ANZ; approved on November 9, 2023, chaired by Prof. Dr. Carola Seifart). Participants accessed the survey via a link that directed them to the study’s homepage on the SurveyMonkey platform. The survey began with a brief introduction outlining the purpose and objectives of the study, followed by a consent declaration. Only participants who provided informed consent by actively selecting the acceptance option were able to proceed to the questionnaire. Thus, informed consent to participate was obtained from all participants. This manuscript adheres to the current Strengthening the Reporting of Observational Studies in Epidemiology (STROBE) guidelines.

### Study design

This anonymous web-based survey was conducted among members of the Austrian Society of Anesthesiology, Resuscitation and Intensive Care Medicine (ÖGARI) between January 2024 and February 2024. On January 10, 2024, a newsletter invitation was sent to 1,839 ÖGARI members, requesting participation in the survey. The survey consisted of 32 questions and a brief introduction outlining the rationale and objectives of the survey. Participation was voluntary, with no financial or other incentives provided. Web cookies were used to limit responses to one per participant. The order of the questions remained consistent for all participants. The 32 questions included 30 multiple-choice questions (24 single-answer, six multiple-answer) and two matrix questions with answers based on a Likert scale. In specific cases, subsequent questions were displayed only if the previous response met predefined criteria. The questionnaire used in this survey is provided as Supplementary Material.

### Data management and analysis

Data were managed and analyzed using Microsoft Excel 2013 (Microsoft Corporation; Redmond, WA, USA). Only fully completed questionnaires were included in the analysis. To proceed through the questionnaire, participants were required to answer all preceding items. In line with the guidelines of the American Association for Public Opinion Research (AAPOR) for opt-in online surveys, a formal response rate was not calculated, as the total number of individuals who received and meaningfully engaged with the invitation could not be reliably determined. Instead, we report the absolute number of responses at each stage to maintain transparency.

## Results

Of the 1,839 ÖGARI members who received the email invitation, 201 participated in the survey (11% of all ÖGARI members). Of the 201 submitted questionnaires, 24 were excluded from analysis because they were discontinued around the midpoint of the survey. Key sections on target MAP, vasopressor use, fluid therapy, and hemodynamic targets remained unanswered. As the discontinuations occurred progressively rather than thematically, the missing data were classified as missing completely at random (MCAR) [4]. Consequently, 177 fully completed surveys were included in the final analysis. Table 1 presents data on the respondents’ clinical experience, job positions, hospital settings, and demographics.

**Table 1:** Data on the respondents’ demographics, clinical experience, positions, and current hospital

	*N*	%
Professional experience		
< 5 years	38	21.5
5–10 years	51	28.8
11–20 years	44	24.9
> 20 years	44	24.9
Job position		
Resident	51	28.8
Consultant	29	16.4
Senior Consultant	77	43.5
Chief Physician	18	10.2
Other Position	2	1.1
Workplace/Hospital of the respondents		
Primary Care Hospitals (≤ 299 beds)	3	1.7
Standard Care Hospitals (300–499 beds)	48	27.1
Specialized Care Hospitals (500–799 beds)	54	30.5
Maximum Care Hospitals (≥ 800 beds)	69	39.0
Other Workplace	3	1.7

### Knowledge of the German guideline for intraoperative hemodynamic monitoring and management

Among the 177 fully completed questionnaires, 38% (68/177) of respondents reported being familiar with the content of the new German Guideline on intraoperative hemodynamic monitoring and management in adults undergoing non-cardiac surgery. In contrast, 62% (109/177) stated that they were not familiar with the guideline’s content.

### Perioperative blood pressure measurement

**Fig. 1:**
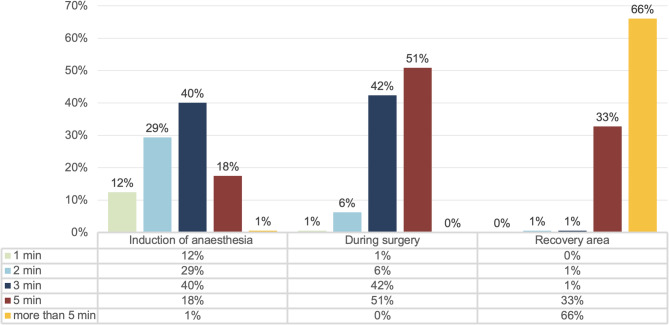
Depiction of measurement intervals for intermittent oscillometric blood pressure monitoring during anesthesia induction, during surgery, and in the recovery area (177 responses each)

When using intermittent oscillometry for blood pressure measurement, respondents reported varying measurement intervals depending on the clinical phase. During anesthesia induction 40% (71/177) measure blood pressure every three minutes, while 51% (90/177) adopted a five-minute interval during surgery. In the post-anesthesia care unit (PACU), 66% (117/177) extended the interval to more than five minutes (see Fig. 1).

**Fig. 2:**
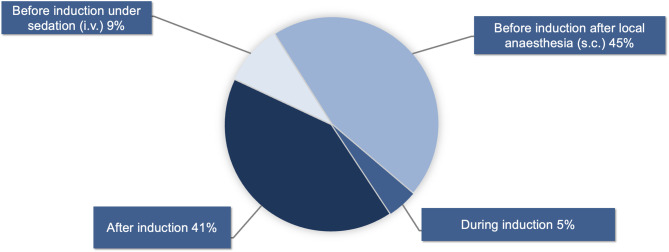
Distribution of arterial catheter insertion time points relative to the perioperative phase. The data illustrate the frequency of insertion before, during, and after anesthesia induction (177 responses)

When an arterial catheter is used to measure blood pressure, 45% (80/177) of respondents routinely insert the catheter before induction of anesthesia after local anesthesia, while 9% (16/177) insert it before induction of anesthesia under intravenous sedation. 5% (8/177) routinely insert it during induction of anesthesia, whereas 41% (73/177) routinely insert the arterial catheter after induction of anesthesia (see Fig. 2).

Regarding invasive blood pressure (IBP) monitoring techniques, 97% (172/177) of respondents reported using direct arterial cannulation comparable to peripheral IV access as their primary method. In contrast, 3% (5/177) prefer the Seldinger technique (catheter over guidewire). All respondents (100%, 177/177) insert arterial catheters in the radial artery. 11% (19/177) of the respondents “always” use ultrasound for arterial catheter insertion, while 33% (58/177) use it only after the “first failed attempt” and 35% (62/177) after “multiple failed attempts”. 21% (35/177) use ultrasound “sometimes”, while 1% (3/177) “never” use ultrasound for arterial catheter insertion.

To assess the quality of the arterial pressure waveform (e.g., for over- or underdamping), 75% (133/177) of the respondents rely on visual inspection of the waveform, while 19% (33/177) perform a square wave test (fast flush test, eyeballing). 5% (8/177) do not routinely check the arterial pressure waveform quality, and 2% (3/177) use other methods.

For patients undergoing surgery in the sitting position, 31% of the respondents (54/177) position the pressure transducer at the level of the ear canal, whereas 65% (115/177) place it at heart level. An additional 5% (8/177) selected ‘other’ positioning methods.

Regarding blood pressure management, nearly all respondents (95%, 168/177) use mean arterial pressure (MAP) as the primary parameter, while 5% (9/177) rely on systolic blood pressure. Diastolic blood pressure is never used (0%, 0/177).

**Fig. 3:**
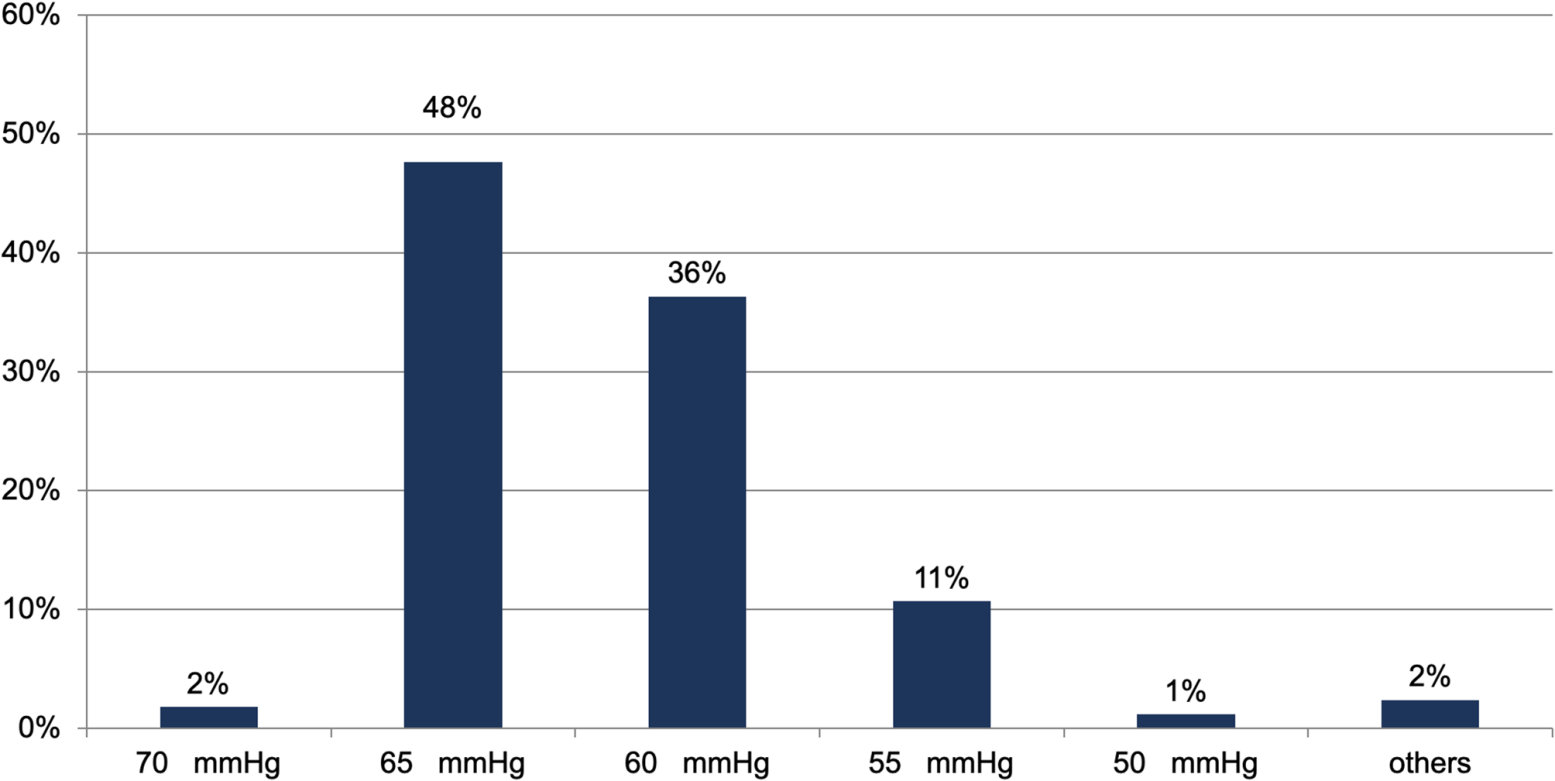
Distribution of MAP thresholds considered critically low by participants, based on 177 responses. The figure illustrates the variability in clinicians' perceptions of critical hypotension during the perioperative period

Among those using MAP to Guide blood pressure management, opinions on critical MAP thresholds varied. 36% (61/168) define 60 mmHg as “critically low”, while 48% (80/168) define 65 mmHg as “critically low” (see Fig. 3). When addressing intraoperative hypotension, 79% (140/177) reported managing it at their own discretion. 9% (16/177) follow the instructions of a consultant anesthesiologist, while 12% (21/177) adhere to a standardized institutional protocol.

### Availability and use of vasoactive agents

**Fig. 4:**
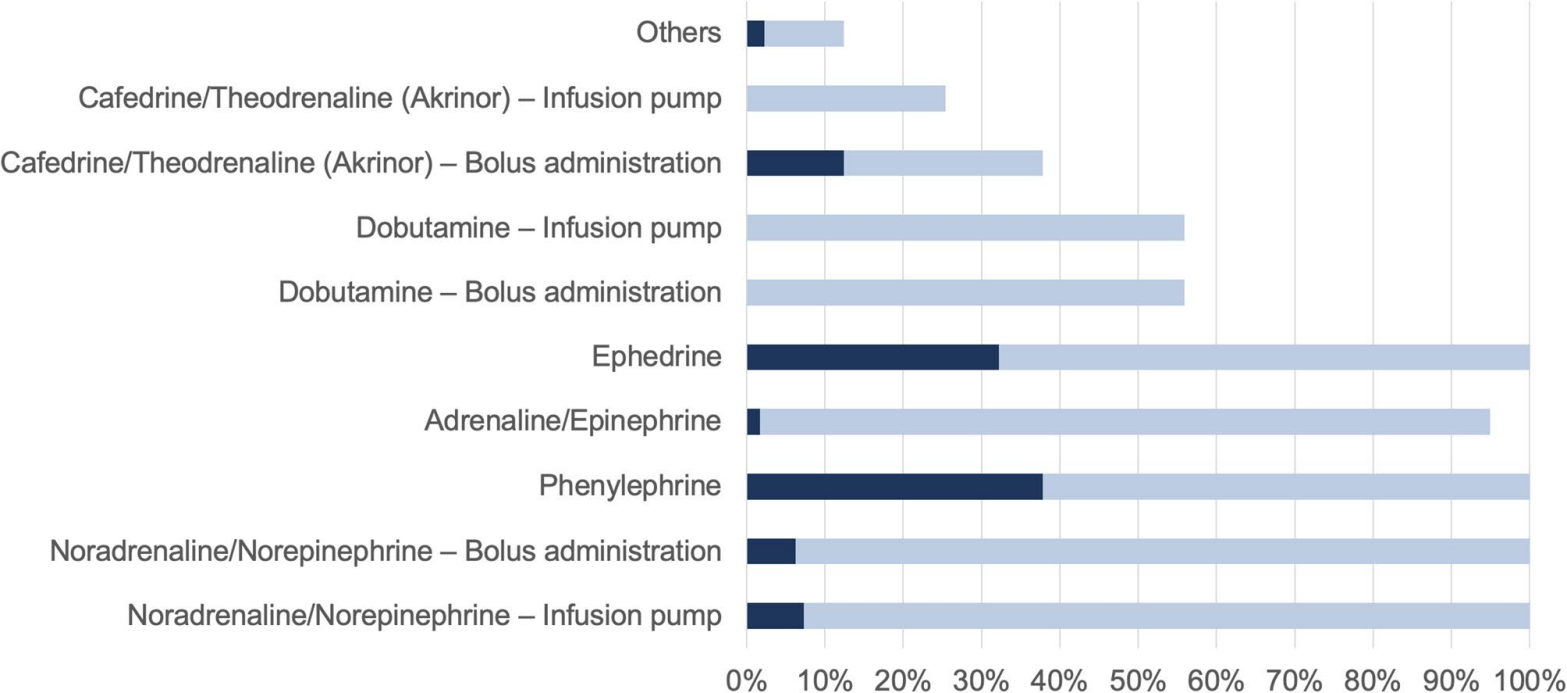
Availability and use of vasoactive drugs: The light blue bars indicate the proportion of respondents who reported that the respective agent is generally available in the OR for the treatment of intraoperative hypotension (multiple selections allowed). The dark blue bars represent the proportion of respondents who selected the respective drug as their preferred choice for managing anesthesia-induced hypotension (177 responses)

Participants were asked which vasoactive agents were typically available in the operating room (OR) for the treatment of intraoperative hypotension (multiple selections allowed). Respondents were also asked to identify the most commonly used drug to treat anesthesia-induced hypotension and achieve target blood pressure in patients undergoing non-cardiac surgery (see Fig. 4).

## Advanced hemodynamic monitoring

The majority of respondents reported “always/frequently” considering patient comorbidities (92%, 162/177), surgical risk (90%, 159/177), expected blood loss (84%, 148/177) and the American Society of Anesthesiologists physical status classification (70%, 123/177) when determining the need for advanced hemodynamic assessment.

The duration of surgery was less consistently considered, with 51% (90/177) of respondents “occasionally” taking it into account and 10% (18/177) “rarely or never” considering it a determining factor. The most commonly used methods for advanced hemodynamic monitoring in the respondents’ hospitals are presented in Table 2.

**Table 2:** Methods used for advanced hemodynamic monitoring and their frequency of use in Austrian hospitals (‘frequently’, ‘rarely’, ‘never’, ‘not available’). Data are shown as percentages with absolute numbers in parentheses

	frequently		rarely		never		technology not available	
Pulse contour analysis (*n* = 177)	49%	(87)	40%	(71)	5%	(9)	6%	(10)
CVP monitoring (*n* = 177)	43%	(76)	39%	(69)	17%	(30)	1%	(2)
TTE (*n* = 177)	42%	(74)	36%	(63)	18%	(32)	5%	(8)
TEE (*n* = 177)	22%	(39)	50%	(88)	21%	(38)	7%	(12)
Transpulmonary thermodilution (*n* = 177)	18%	(32)	48%	(85)	21%	(38)	12%	(22)
Pulmonary artery catheter (*n* = 177)	7%	(13)	38%	(67)	34%	(61)	20%	(36)
Oesophageal Doppler (*n* = 177)	6%	(11)	23%	(40)	27%	(47)	45%	(79)
Finger cuff technology (*n* = 177)	4%	(7)	21%	(37)	18%	(32)	57%	(101)
Bioreactance/Bioimpedance technology (*n* = 177)	3%	(6)	11%	(20)	20%	(35)	66%	(116)

*CVP* Central venous pressure, *TEE* Transesophageal echocardiography, *TTE* Transthoracic echocardiography

### Perspectives of hemodynamic monitoring

When asked about potential reasons for the limited use of advanced hemodynamic monitoring in high-risk non-cardiac surgery patients, respondents reported several factors. 52% (92/177) cited the time-consuming setup, while 64% (113/177) perceived the added value as too low. A lack of experience in interpreting hemodynamic parameters was reported by 57% (100/177), whereas 23% (40/177) considered the cost of monitors and disposables a limiting factor. Additionally, 29% (51/177) indicated that not enough monitors are available. Regarding the potential benefits of advanced hemodynamic monitoring, 41% (73/177) believed that it “sometimes” improves patient care in the OR, while 57% (101/177) considered its impact to be “(almost) always” beneficial.

### Assessment of fluid responsiveness and acute blood loss management

When assessing fluid responsiveness in patients with sinus rhythm and controlled mechanical ventilation, 50% (88/177) of the respondents rely on visual interpretation of the arterial pressure waveform (“swing”). Additionally, 33% (59/177) use a “fluid challenge” (250–500 ml), while 34% (60/177) consider heart rate as an indicator.

Regarding the type of fluids used for fluid challenges, the majority 89% (158/177) administer balanced crystalloid fluids, while 5% (8/177) prefer gelatin-based solutions. Only 3% (5/177) use NaCl 0.9%, 2% (4/177) prefer other fluids, and 1% (2/177) administer hydroxyethyl starch (HES).

As part of the survey, participants were presented with a clinical scenario involving a patient experiencing acute intraoperative blood loss and hypotension following the administration of 750 ml of isotonic electrolyte solution over five minutes. When asked to select their preferred fluid therapy until blood products (red blood cells, fresh frozen plasma, platelet concentrates) became available, 49% (87/177) of respondents opted for gelatin-based colloids, while 27% (48/177) preferred a balanced electrolyte solution. Additionally, 20% (35/177) chose albumin, whereas 2% (3/177) reported using HES. None of the respondents (0%, 0/177) indicated using dextrans or 0.9% NaCl, while 2% (4/177) selected ‘other’ solutions.

## Discussion

This web-based survey among ÖGARI members provides valuable insights into current hemodynamic monitoring and management practices during non-cardiac surgery in Austria. The findings suggest that respondents largely follow international recommendations, particularly concerning general blood pressure thresholds, measurement intervals, and indications for advanced hemodynamic monitoring. However, hemodynamic and blood pressure management appear to be only partially standardized, with decisions primarily left to the discretion of the anesthetist.

The results also indicate a certain level of awareness of the recently published German guideline on ‘Intraoperative hemodynamic monitoring and management in adults undergoing non-cardiac surgery’ within the Austrian anesthesiology community [2]. A notable proportion of respondents reported familiarity with its content just a few months after its release, suggesting increasing recognition and engagement with this topic.

In most patients, blood pressure monitoring during surgery is performed using intermittent oscillometric measurements. During anesthesia induction, many respondents measure blood pressure every two to three minutes [5–7]. However, during surgery, the most commonly reported measurement intervals are longer, usually three or five minutes, likely due to anesthetists’ experience that induction of anesthesia is often accompanied by hypotension [7]. In the PACU, measurement intervals are often extended beyond five minutes, presumably due to greater hemodynamic stability [8].

When intra-arterial blood pressure monitoring is indicated, more than one-third of the respondents insert arterial catheters only after anesthesia induction, possibly to enhance patient comfort. However, pre-induction arterial catheter placement reduces the incidence of hypotension and potentially prevents organ injury [9].

Moreover, similar procedures, such as arterial access during cardiac catheterization, are routinely performed in awake patients, suggesting that pre-induction placement may be a feasible and beneficial approach. The findings indicate a strong preference for direct arterial cannulation as the primary method for invasive blood pressure monitoring, while the Seldinger technique is used only occasionally. However, studies in neonates, infants, and adults suggest that the guidewire-assisted technique (Seldinger technique) achieves higher first-attempt success rates and lower complication rates, particularly in challenging anatomical conditions [10, 11]. This discrepancy suggests that the choice of technique is influenced by clinician expertise and patient-specific factors, with direct puncture remaining a cost-effective standard option. Compared to their Austrian counterparts, German clinicians more frequently perform arterial cannulation using Seldinger technique into routine practice [3].

Ultrasound devices were generally available for advanced hemodynamic monitoring. However, this availability did not consistently translate into the use of ultrasound guidance for arterial catheter placement. This discrepancy underscores a relevant gap between technological availability and its routine clinical application, despite well-established benefits of ultrasound-guided arterial access [12, 13].

Assessing arterial pressure waveform quality is crucial, particularly in the context of pulse wave analysis [14, 15]. While approximately 75% of respondents rely on visual inspection to detect damping phenomena in the blood pressure curve, only 19% employ standardized tests [14, 16]. These findings indicate that visual assessment remains the predominant method for evaluating arterial pressure waveforms. Given the critical role of waveform accuracy in pulse wave analysis, implementing standardized testing methods, such as the fast-flush test, may enhance the reliability of hemodynamic monitoring in clinical practice.

In addition to continuous invasive blood pressure measurement, continuous non-invasive monitoring via finger cuff technology may contribute to reducing intraoperative hypotension [17, 18]. However, more than half of the respondents reported lacking access to such monitoring systems, and only 4% indicated frequent use of this technology. These findings suggest that finger cuff technology is not yet routinely integrated into clinical practice among the respondents. Compared to survey data from Germany, finger cuff monitoring appears less frequently adopted in Austrian hospitals, although its overall utilization remains limited in both countries [3].

Furthermore, participants were asked about the use of standardized protocols for the treatment of intraoperative hypotension. With only one in ten respondents following such protocols, blood pressure management appears largely unstandardized, which may lead to variability and reduced reproducibility [19]. Although protocol-based approaches may help improve blood pressure stability and [20], when combined with machine learning tools, significantly reduce the incidence and duration of hypotension [21], robust evidence for improved patient outcomes remains limited [22, 23]. Standardized protocols, tailored to local clinical structures and resources, may represent a pragmatic approach to improving consistency and quality in blood pressure management. Compared to the Austrian results, the German survey data indicate greater adherence to standardized treatment pathways, with less reliance on individualized clinical decision-making in the management of intraoperative hypotension [3].

Large database studies have demonstrated an association between perioperative hypotension and postoperative organ injury [6, 24–26]. Although no universally accepted definition of hypotension exists [27, 28], it is commonly defined as a MAP below 60–65 mmHg – a threshold associated with an increased risk of acute kidney and myocardial injury [28, 29]. Consistent with these findings, a substantial proportion of respondents manage blood pressure based on MAP and consider a MAP of 60–65 mmHg critically low. Raising alarm thresholds to reflect these critical MAP values may help reduce hypotension episodes and support timely intervention. This simple adjustment represents a pragmatic and easily implementable strategy to enhance intraoperative blood pressure management in everyday practice [30].

The survey results also reflect the availability of standard vasoactive agents for intraoperative hypotension management in Austria. Phenylephrine and ephedrine were the most frequently used agents, consistent with the findings of Baekgaard et al., who identified both substances among the most commonly employed vasoactive drugs in clinical trials for hypotension treatment [31]. Cafedrine/Theodrenaline (Akrinor^®^), available to only 25% of respondents, was the third most frequently used agent, despite its limited availability outside Germany [32].

According to current recommendations [33], respondents select advanced hemodynamic monitoring based on patient comorbidities, surgical risk, and expected blood loss. While these considerations appear appropriate, studies consistently indicate that advanced hemodynamic monitoring remains underutilized in surgical patients, including those at high risk [34, 35].

To identify potential barriers to implementation, respondents were asked to specify contributing factors. A substantial proportion of respondents perceived the added value of advanced hemodynamic monitoring as too low, while more than half reported a lack of experience in interpreting hemodynamic parameters. Despite the widespread availability of pulse wave analysis in clinical settings, only a minority of respondents reported regular use. These findings highlight a considerable gap between the availability and clinical utilization of advanced hemodynamic monitoring, aligning with findings from a recent survey among German anesthesiologists [3].

The limited uptake may reflect ongoing uncertainty regarding appropriate indications, therapeutic targets, and the lack of compelling outcome evidence. Recent meta-analyses have shown that goal-directed hemodynamic therapy (GDHT) using parameters such as stroke volume variation or cardiac output can reduce postoperative complications in high-risk patients undergoing major non-cardiac surgery [36–38]. In contrast, large randomized trials, specifically the iPEGASUS trial by Funcke et al. and the OPTIMISE II trial conducted by the OPTIMISE II Trial Group, found no benefit in actively maintaining an optimized postinduction cardiac index [22, 23]. Notably, both studies reported an increased incidence of adverse cardiac events. These conflicting findings likely contribute to clinician skepticism regarding the routine use of advanced monitoring.

The frequently reported insufficient familiarity with advanced hemodynamic parameters underscores the need for structured training and continuous education. A solid grasp of cardiovascular physiology and monitoring principles is essential for accurate interpretation [39], as misinterpretation may lead to inappropriate treatment and compromise patient safety [40, 41]. Several clinical studies have investigated educational strategies to improve competency in this area.

High-fidelity simulators combined with advanced monitoring systems have proven effective in teaching cardiovascular physiology to anesthesia providers [42, 43]. Simulation-based models have also been successfully used to train critical care physicians in advanced ventilation and hemodynamic management [44]. Notably, medical learners who observed their own real-time hemodynamic parameters during personalized simulation scenarios demonstrated a significant improvement in understanding complex physiological concepts [45]. These methods allow realistic clinical training without exposing patients to risk. To further enhance clinical application, structured interpretation tools such as validity checklists have been proposed to support clinical decision-making and promote appropriate use of advanced hemodynamic parameters [46].

A follow-up study is planned for late 2026. It will revisit the present key findings, including the use and further development of local protocols, potential improvements in the interpretation of advanced hemodynamic parameters, and the clinical application of continuous non-invasive monitoring. To broaden the perspective, an international extension via the European Society of Anaesthesiology and Intensive Care is being considered.

### Strengths and limitations

This study is based on 177 fully completed responses, providing a differentiated view of current hemodynamic monitoring and management practices during non-cardiac surgery in Austria. The sample was exclusively composed of medical professionals, with negligible participation from non-physician staff. A high proportion of respondents held senior clinical positions, enhancing the clinical validity and interpretability of the findings. In addition to structural parameters, the survey assessed clinical decision-making with regard to indication thresholds, hemodynamic targets and pharmacological interventions. Many key findings of this survey among Austrian anesthesiologists are consistent with the results of the nationwide German survey and reveal comparable trends and barriers in clinical routine in both countries.

The study is not without limitations.

First, the response rate was 11% of all contacted ÖGARI members. While this figure is consistent with other anonymous online surveys conducted without incentives or reminders, it limits generalizability. ÖGARI includes not only anesthesiologists but also nursing staff, associate members, and physicians working solely in intensive care. These groups may not have felt addressed by a survey on intraoperative hemodynamic management, potentially contributing to limited participation.

A comparable survey on advanced hemodynamic monitoring among members of the German Society of Anaesthesiology and Intensive Care Medicine (DGAI), also with a limited response rate, was recently published by our group [3]. While the response rate was acknowledged as a limitation, publication in a peer-reviewed journal underlined that scientific value is primarily determined by the relevance of the research question, methodological transparency and the professional qualification of respondents.

Furthermore, Austria’s healthcare system is characterized by a high degree of centralization, in contrast to more decentralized systems such as Germany. Specialized services, such as advanced hemodynamic monitoring, are typically concentrated in high-capacity university or tertiary hospitals. This may have also contributed to the limited participation rate.

Second, voluntary participation may have led to a predominance of respondents with a specific interest in hemodynamic monitoring and access to advanced technologies. This introduces the risk of selection bias and may lead to an overestimation of the frequency of advanced monitoring modalities.

Third, the survey exclusively captured physician perspectives. The views of critical care nurses - particularly relevant in intensive care settings - were not included. Anesthetists working in outpatient or day-case settings, where advanced monitoring is less commonly applied, may have been underrepresented.

Fourth, as with all self-reported data, discrepancies between stated practices and actual clinical behavior must be considered. This applies in particular to questions regarding protocol adherence and monitoring indications.

Fifth, although technical measures (e.g., cookie-based response blocking) were employed to prevent multiple submissions, repeated participation by individual respondents cannot be ruled out entirely.

Sixth, due to the anonymous nature of the survey, it cannot be determined whether multiple respondents originated from the same hospital. This could potentially lead to an overrepresentation of local clinical standards in the results, particularly if these respondents followed the same standardized protocols. We acknowledge that hospital-specific protocols might contribute to response variability. However, the data indicate that intraoperative blood pressure management was predominantly guided by individual clinical judgment rather than by institutional protocols. Therefore, the influence of potential institutional clustering on the overall findings is likely limited.

Despite these methodological constraints, the present survey provides a robust and clinically relevant snapshot of perioperative hemodynamic monitoring and management practices in Austria - particularly within tertiary care centers and highly specialized surgical environments.

## Conclusion

Our survey among ÖGARI members provides valuable insights into intraoperative hemodynamic monitoring and management in Austrian hospitals. While key aspects of blood pressure monitoring and management align with current recommendations, our findings suggest that blood pressure management largely remains at the discretion of individual clinicians rather than being guided by standard operating procedures (SOPs). Despite the availability of cardiac output monitors, fewer than half of the respondents use them regularly. A key barrier to the wider adoption of advanced hemodynamic monitoring appears to be a lack of specialized expertise in interpreting hemodynamic parameters. These findings highlight the need for targeted education and training programes in perioperative hemodynamic monitoring.

## Supplementary Information

Supplementary Material 1.

## Data Availability

The datasets generated and analyzed during the current study are available from the corresponding author on reasonable request.

## Abbreviations

AAPOR: American Association for Public Opinion Research
ASA: American Society of Anesthesiologists
CO: Cardiac Output
CVP: Central Venous Pressure
DGAI: German Society of Anaesthesiology and Intensive Care Medicine
DRKS: German Clinical Trials Register
HES: Hydroxyethyl Starch
IBP: Invasive Blood Pressure
MAP: Mean Arterial Pressure
OR: Operating Room
PACU: Post-Anesthesia Care Unit
SOP: Standard Operating Procedure
STROBE: Strengthening the Reporting of Observational Studies in Epidemiology
TEE: Transesophageal echocardiography
TTE: Transthoracic Echocardiography
ÖGARI: Austrian Society of Anesthesiology, Resuscitation and Intensive Care Medicine
